# Female Sexual Dysfunction in Association with Sexual History, Sexual Abuse and Satisfaction: A Cross-Sectional Study in Hungary

**DOI:** 10.3390/jcm12031112

**Published:** 2023-01-31

**Authors:** Csaba Erdős, Oguz Kelemen, Dávid Pócs, Edina Horváth, Nóra Dudás, András Papp, Edit Paulik

**Affiliations:** 1 Department of Public Health, Albert Szent-Györgyi Medical School, University of Szeged, 6720 Szeged, Hungary; 2 Department of Behavioural Sciences, Albert Szent-Györgyi Medical School, University of Szeged, 6720 Szeged, Hungary; 3 Family Medicine Department, University of Szeged, 6725 Szeged, Hungary; 4 Department of Psychiatry, Ödön Jávorszky Hospital, 2600 Vác, Hungary

**Keywords:** female sexual dysfunction, DSM-5, sexual disorder, sexual abuse, sexual history, satisfaction

## Abstract

Female sexual dysfunction (FSD) has a negative impact on women’s quality of life, self-esteem, and physical health. The aim of the present study was to explore the prevalence and the factors associated with FSD using an online questionnaire. A cross-sectional survey was conducted among young adults (18−35 years old) in Hungary using the DSM-5 criteria. The participants (*n* = 5942) were divided into three major groups: FSD (20.3%), an intermediate group (43.9%), and a control group (35.6%). Most of the women showing FSD were affected with female orgasmic disorder (9.2%) and genito-pelvic pain/penetration disorder (4.6%), while female sexual interest/arousal disorder was found in altogether 100 women (1.7%); 4.8% of women were affected by more than one definite disorder. The occurrence of female sexual dysfunction was related to the women’s previous sexual history (first sexual experience, sexual education, early encounter with pornographic content, and sexual abuse), their self-satisfaction (with their own body, genitalia, and sexual attraction), and their sexual orientation. Sexual dysfunction showed a strong association with abuse, sexually transmitted diseases, and self-esteem. The present study identified the relationship between sexual dysfunctions and other health conditions, which can be the basis for some form of screening and early assistance programs for FSD.

## 1. Introduction

Female sexual dysfunction (FSD) is a heterogeneous group of multi-causal and multi-dimensional medical disorders that adversely affect physical health and emotional well-being [1]. FSD is a common condition in women of all ages and has a negative impact not only on their quality of life but also on the sexual function and quality of life of their partners. As the condition can affect women’s mental health, an impact on the whole family and society cannot be excluded either [2].

Goldstein et al. found that low sexual desire is a common but often undiagnosed condition [3]. Their study revealed a relationship between low sexual desire and lower quality of life, psychosocial factors, such as dissatisfaction with having sex with a partner or within the marriage, and negative emotional states, including frustration, hopelessness, anger, low self-esteem, loss of femininity, self-reported poor health, as well as depression and anxiety [3]. Stephenson et al. found that women who had been sexually abused during their childhood later expressed concern about their sexual function [4]. Better sexual function was associated with better-rated body image and longer-lasting relationships, whereas poorer sexual function showed the opposite [5]. Similarly, Reissing’s research showed lower self-esteem in women with vaginismus [6]. Pazmany et al. found that dyspareunia negatively affected the women’s body image and genital self-esteem, eventually leading to sexual anxiety [7].

The etiology of FSD is complex and has a multifactorial background, where psychological, social, and biological factors play an important role [6,7,8,9,10]. One of the most cited psychosocial factors is abuse. Childhood sexual abuse has been shown to be positively associated with vaginismus, lubrication failure, orgasmic disorder, and female sexual interest/arousal disorders [4,6]. It is of particular interest that the Iranian sample in the study of Safarinejad is also characterized by previous and current sexual assault and sexual harassment resulting in diminished sexual desire [9]. According to the research of Brassil and Keller, sexual dysfunction is strongly related to early sexual experiences [10].

In various studies, the prevalence of FSD among sexually active women is between 30 and 50%. [11,12,13]. According to the consensus of the 4th International Consultation on Sexual Medicine (ICSM) in 2015, the prevalence of at least one sexual dysfunction among women, regardless of their age, is approximately 40–50% [11]. The results from a global study of 27,500 people (about half of whom were women aged 40–80 years) showed that a large proportion of women experienced multiple sexual dysfunctions: 17% in Northern Europe and 34% in Southeast Asia [12]. In Slovenia, the prevalence of women with at least one sexual dysfunction is 31% [13].

In 2013, the fifth edition of the American Psychiatric Association’s Diagnostic and Statistical Manual of Mental Disorders (DSM-5) revised the criteria for FSD [14]. The previous categories of hypo-active female sexual desire disorder and female sexual arousal disorder were merged into the single diagnosis of female sexual interest/arousal disorder (FSIAD). Dyspareunia and vaginismus were also merged into a single diagnosis of genito-pelvic pain/penetration disorder (GPPPD). Sexual aversion disorder was eliminated completely [15].

Prevalence rates across female sexual dysfunctions differ by context and across DSM editions/definitions, which is problematic because it makes it difficult to introduce integrated prevalence data. Of the categories used currently, only female orgasmic disorder (FOD) was included in the previous version of DSM (DSM-IV). The estimated prevalence of FOD varies between 20 and 40% internationally [16]. A Mexican study found an 18.3% prevalence of FOD between 18–40 years of age in Mexico [17]. Orgasmic disorder has been reported to be prevalent at 16–25% in Australia, the United States, Canada, and Sweden, with a higher prevalence of 37% in Iran [11]. In Northern Europe, the prevalence of orgasmic dysfunction was 10–12% between 18–34 years [16]. According to a study of adults aged 40–80 conducted in 29 countries, orgasmic disorders are slightly more common among women in Asian countries [17].

Since the release of the DSM-5, data about FSIAD have been barely available. Earlier findings were based on the terms hypoactive sexual desire disorder (HSDD) and female sexual arousal disorder (FSAD), used in DSM-IV-TR. A study reported low sexual interest in 22% of women in the general US population [18]. Lack of sexual interest was the most common sexual problem in Southeast Asia [11]. The prevalence studies of sexual arousal disorders in women focused primarily on the lack of vaginal lubrication. These studies found that 8–15% of all women and 21–28% of sexually active women report such complaints [19].

The prevalence estimates of GPPPD are heterogeneous because of the variations in diagnostic criteria, methods, and study design. Earlier studies using previous DSM concepts (mainly the DSM-IV) concluded that the prevalence rates in the general population varied between 3 and 25% for dyspareunia and 0.4 and 6.6% for vaginismus [20,21,22,23]. Dyspareunia affected 2% of elderly British women [24].

As no study has addressed the prevalence of FSD in Hungary since the release of the DSM-5, the main goal of the current study was to explore the prevalence and the factors associated with female sexual dysfunction among young adults using an online questionnaire based on DSM-5 as a theoretical framework.

## 2. Materials and Methods

**Figure 1:**
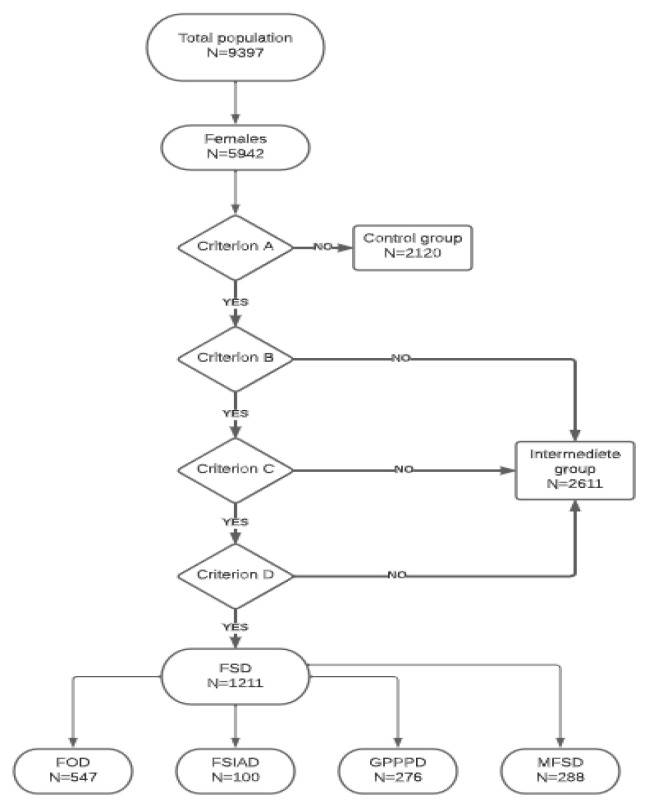
Transformation of DSM-5 criteria into online questions. The DSM-5 identifies three female sexual disorders (FOD, FSIAD, GPPPD) within sexual dysfunctions. The DSM-5 is structured by dividing each criterion into different subsections: Criterion A is the presence of the symptom. Criterion B is duration, that is, how long the symptoms persist. Criterion C draws attention to the fact that symptoms cause clinically significant suffering to the individual (according to subjective acknowledgment), thus attempting to avoid an excessive tendency to medicalization. Criterion D, as differential diagnostic issues are sequenced and excluded (the problem is not better explained by a nonsexual mental disorder and cannot be attributed to causes such as relationship stressors, medical conditions, or medication/substance effects). FSD: Female Sexual Dysfunction. FOD: Female Orgasmic Disorder. FSIAD: Female Sexual Interest/Arousal Disorder. GPPPD: Genito-Pelvic Pain/Penetration Disorder. MFSD: Multiple Female Sexual Dysfunction. DSM-5: Diagnostic Statistic Manual of Mental Disorders 5th edition.

The cross-sectional study was conducted by means of an online web-based questionnaire between September and December 2018 in Hungary. A convenience sampling technique was applied, and the participants were recruited through the webpage of the Medical Faculty of the University of Szeged and by promotions on various social media platforms (Facebook and Instagram). The target population was young adults (18–35 years old). Those who gave unrealistic, frivolous, or inappropriate answers (e.g., unrealistic body weight and height, unrealistic number of sexual partners related to age, etc.) were removed from the database. Finally, 9397 individuals participated in our study, of which 6367 were women based on their biological sex. Due to the use of an online method, we had no control over the age of the respondents, so 6% were over 35 years old and excluded from the present analysis. The final number of analyzed young adult women was 5942 (Figure 1).

### 2.1. Data Collection

#### 2.1.1. Transformation of the DSM-5 Criteria into Online Questions

DSM-5 identifies three female sexual disorders (FOD, FSIAD, GPPPD) within FSD [14]. The participants of our study were divided into three major groups according to the DSM-5 A-B-C-D criteria. Figure 1 shows the flowchart of our classification. The control group consisted of those who answered no to DSM-5 criterion A (experience of symptoms). Respondents who answered ‘Yes’ to criterion A but ‘No’ to criteria B, C, and D formed the intermediate group; these subjects remained in the database but were not included in the present analysis. Respondents who met all four criteria formed the group of women with FSD. In their case, we analyzed separately those who had only one type of FSD (FOD, FSIAD, GPPPD) and those who had more than one female sexual disorder simultaneously (multiple FSD, MFSD). The FSD group included women affected by a disorder according to the DSM-5 criteria system (A, B, C, or D). Within this, we analyzed the different disorders one by one (FOD, FSIAD, GPPPD) and MFSD. The online questions were structured in such a way that questions eliciting an affirmative reply were followed by more detailed questions to assess the recognition; however, if we received a negative answer, the additional questions were automatically skipped. The advantage of this method was that the relevant issues were sufficiently explored, and the questionnaire was not discouragingly long.

#### 2.1.2. Variables

The dependent variables included the presence of FOD, FSIAD, GPPPD, and MFSD. The independent variables were related to sexual history, sexual orientation, previous sexual abuse, and sexual self-esteem, as well as age.

- Sexual history was measured by four questions: (1) the satisfaction with the first sexual experience (evaluated by a 1 to 10 scale: 1 = very bad, 10 = very good); (2) previous sexual education (yes–no); (3) the age at the first encounter with pornographic content (age in years); (4) the lifetime prevalence of sexually transmitted diseases (STD) (yes–no).
- Sexual orientation was measured by four categories: heterosexual, homosexual, bisexual, and asexual.
- The sexual self-esteem of women was evaluated by three questions: (1) satisfaction with their own body image (e.g., body weight, height, abdominal circumference, shoulder or waist width, and face); (2) satisfaction with their own genitalia, (3) satisfaction with their own sexual attraction. All three questions were answered on a 1 to 10 scale:1 = not at all satisfied, 10 = completely satisfied.

### 2.2. Statistical Analysis

All analyses were performed with IBM SPSS Statistics 28.0 software. The dependent variables were analyzed in comparison with the control group. The categorical variables were examined with the Pearson Chi-square test. The normality of continuous variables was tested with the Kolmogorov-Smirnov test, and because of the non-normal distribution, these variables were analyzed with the Mann-Whitney U test. The results are presented in the form of percentages, mean values, standard deviation (±SD), and median values. All data with *p* < 0.05 were considered significant.

## 3. Results

The characteristics of the participants are shown in Table 1.

The average age was 22.16 years (SD: ±3.51), 23.5% were single, 8.3% had a sexual partner, 67.6% lived in marriage or a partnership, and 0.8% had more than one relationship simultaneously. The evaluation of the first sexual experience was 5.68 ± 2.65). The mean age when experiencing pornographic content was 14.48 ± 3.16. The majority (69.5%) received sexual education, and 14.2% already had some form of STD. The majority (92.2%) were heterosexual, 1.1% homosexual, 6.3% bisexual, and 0.3% asexual. In total, 13.8% reported experiencing sexual abuse. Satisfaction with their own body showed a mean value of 5.29 ± 2.59; with their own genitalia, 7.34 ± 2.43; and with their own sexual attraction, 6.08 ± 2.08.

### 3.1. Factors Associated with FOD

Sexuality-related characteristics of women affected by FOD (*n* = 547, 9.2%) and the control group are shown in Table 2. The mean age of women in the FOD group was 22.12 ± 3.41 years, whereas, in the control group, it was 22.26 ± 3.64 years (*p* = 0.720). There was a significant difference (*p* < 0.001) in the evaluation of the first sexual experience within their sexual history: women in the FOD group evaluated their first sexual experience significantly worse than the control group.

There was a significant difference in the average age on exposure to the first pornographic content; the mean age of the FOD group was significantly lower than that of the control group (14.01 vs. 14.64), the effect size was small (r = 0.09) (Table 2). The levels of satisfaction were significantly lower in the case of their own sexual attraction (6.03 vs. 6.92), their own body image (4.93 vs. 5.79), and their own genitalia (6.78 vs. 7.99) in the FOD group compared to the control group (*p* < 0.001).

A significantly lower proportion of women in the FOD group received sexual education (64.0% vs. 73.4%) than the control group. A higher proportion of women in the FOD group (18.8%) had some kind of STD during their life, significantly more than in the control group (12.7%, *p* < 0.001). Significantly (*p* < 0.001) more women with FOD (16.5%) experienced sexual abuse during their lifetime than women in the control group (10.8%). There was no noteworthy difference in the sexual orientation of the two groups (Table 1).

### 3.2. Factors Associated with FSIAD

Sexuality-related characteristics of women with FSIAD (*n* = 100; 1.7%) and the control group are shown in Table 3. The mean age of women in the FSIAD group was 21.67 ± 3.19 years, while in the control group, it was 22.26 ± 3.64 years.

The evaluation of the first sexual experience was worse (5.53 ± 2.55) in the FSIAD group than in the control group (6.03 ± 2.55). The level of self-satisfaction was significantly lower in the FSAID group in comparison to the control group in all three aspects, such as sexual attraction (6.23 vs. 6.92), body image (4.79 vs. 5.79), and evaluation of their own genitalia (6.75 vs. 7.99).

Significantly fewer women received sexual education in the FSIAD group (59.0%) vs. the control (73.4%, *p* = 0.002). In terms of STD, 16.0% of the FSIAD group and 12.7% of the control group had a positive history (*p* = 0.011). A higher proportion of women in the FSAID group experienced sexual abuse (19.0%), compared with the control group (10.8%, *p* = 0.011) (Table 1).

### 3.3. Factors Associated with GPPPD

Table 4 shows the association between GPPPD (276, 4.6%) and the scale-type independent variables. The mean age of women in the GPPPD group was 22.16 ± 3.47 years, while in the control group, it was 22.26 ± 3.64.

The evaluation of the first sexual experience showed a significant difference between the two groups: the women in the GPPPD group scored an average of 4.92 points, whereas the mean of the control group was 6.03. The level of satisfaction with their own sexual attraction was significantly lower in the GPPPD group than in the control group (6.03 vs. 6.92. The GPPPD group was significantly less satisfied with their own body image and with their own genitalia than the control group (4.93 vs. 5.79, 6.78 vs. 7.99, respectively) (Table 4).

Fewer women received adequate sexual education in the GPPPD group (64.1%) than in the control group (73.4%, *p* < 0.001). A higher proportion of women in the GPPPD group had an STD (18.5%) compared with the control group (12.7, *p* = 0.008). Sexual abuse occurred significantly more frequently in the GPPPD group (19.6%) than in the control group (10.8%, *p* < 0.001) (Table 1).

### 3.4. Factors Associated with MFSD

Altogether 288 women (4.8%) were affected by more than one definite disorder (MFSD group) (Table 5). The mean age of the MFSD group was 22.16 ± 3.47.

The MFSD group found the first sexual experience significantly discouraging compared with the control group (4.92 vs. 6.03). The level of satisfaction was lower in the MFSD group than in the control group in the case of own sexual attraction (5.36 vs. 6.92), own body image (4.32 vs. 5.79), and own genitalia (5.83 vs. 7.99).

More than half (57.3%) of the MFSD group and 73.4% of the control group received adequate sexual education (*p* < 0.001). The proportion of sexual abuse was more than double in the MFSD group (22.2%) compared with the control group (10.8%, *p* < 0.001).

## 4. Discussion

The main aim of this research was to identify the various types of FSD in the young adult female population in Hungary by applying the DSM-5 criteria system. Our results showed that 20.3% of women had some type of FSD according to the criteria of the DSM-5. However, there was a high number (43.9%) of women with sexual complaints who did not meet all criteria of a disorder of DSM-5 and were so classified in the intermediate group.

Our results suggest that female sexual dysfunction is associated with women’s sexual history (of the first sexual experience, presence or absence of sexual education, an early encounter with pornographic content, and sexual abuse), their self-satisfaction (with their own body, genitalia, and sexual attraction) and their sexual orientation. All three conditions (FOD, FSIAD, GPPPD) showed a strong association with abuse, STDs, and self-esteem (self-image).

International studies found a positive correlation between abuse and sexual life, so abuse seems to be an etiological factor of sexual disorders [8,9]. Our research, addressing a wide range of subjects than other studies, found similar results in a self-reported online survey among young people.

The association with self-esteem was investigated by several studies; however, not according to the DSM-5 or not among young adults [7,8]. Brassil and Keller drew attention to the role of early sexual experience in the subsequent development of FSD [10]. Our results, showing a significant association between early sexual experience with FSIAD and GPPPD, are in accordance with their findings.

FOD, as defined in DSM-5, occurred in 547 cases (9.2%) in our study. Our results are comparable to a study by Mexican authors, who used the Female Sexual Function Index (FSFI) and the term orgasmic disorder and found an 18.3% prevalence of FOD [25]. Orgasmic difficulties and anorgasmia affect 11–41% of women worldwide [17,18,19].

Previous studies have found that women with FOD have more negative and fewer positive cognitive associations [26,27,28]. In our study, orgasmic disorder showed a correlation with psychological factors and sexual history. Our results show that satisfaction issues related to self-esteem also correlated with FOD.

DSM-IV-TR had separated definitions of hypoactive sexual desire disorder (HSDD) and female sexual arousal disorder (FSAD) in diagnoses. FSIAD was newly introduced in DSM-5, so no prevalence studies on it have been published yet; most publications measured the prevalence of HSDD and FSAD. In our results, FSIAD occurred in only 100 cases (1.7% of our sample), but our research was conducted among women between the ages of 18 and 35. Further, women (27.4% of the sample belonging to the intermediate group) reported problems with sexual interest, which is similar to international prevalence data. One of the most cited prevalence studies from 1999 reported low sexual interest in 22% of women in the population of the United States [18]. A survey in 2005 covering 29 countries showed self-reported low sexual interest ranging from 26 to 43% [17]. Most research focused on the role of childhood sexual abuse analyzing FSAD and HSDD [29,30]. Our research focused on lifetime (not only childhood) experiences of sexual abuse. In accordance with other studies showing a strong association between the occurrence of sexual abuse and sexual interest disorder, in our research, previous sexual abuse was more common in those who had FSIAD (19%).

Most prevalence research on GPPPD divided data into two separate disorders (vaginismus and dyspareunia) [9,17,19]. GPPPD occurred in 276 cases (4.6%) in our sample according to the standard criteria of the DSM-5. Alizadeh reported that 10.5% of women suffered from GPPPD in Iran according to the criteria of the DSM-5 [31]. Portuguese and British prevalence studies reported 9.8 and 7.5% for dyspareunia [22,32]. Lower satisfaction was strongly correlated with GPPPD in our study. This finding shows similarities with other studies, except that satisfaction in our study, included more than one aspect [31,33]. The occurrence of sexually transmitted diseases was more common among women with GPPPD than among women free of this problem.

A possible limitation of the present study is its reduced representativeness due to convenience sampling. Diagnosis of sexual dysfunction requires a thorough gynecological examination and a detailed medical history, particularly with regard to alcohol and drug use and medication taken. Therefore, our data were not clinically controlled. In addition, the age range limit of 18–35 years old affects the representativeness of our results. Due to the cross-sectional nature of our study, we could not establish causality. In addition, social desirability may have influenced the subjects’ responses.

The strength of the study was the application of the DSM-5 criteria to a large population. Finally, online data collection can facilitate data provision by avoiding cumbersome communication resulting from the taboo/oversensitive nature of the problem.

## 5. Conclusions

This study is the first to measure female sexual disorders according to the DSM-5 criteria in a large population through an online survey in Hungary. A clinical measuring instrument (DSM-5) was transformed into a questionnaire and applied for the purpose of epidemiological investigation. Thus, the population-level measurement of FSD became possible.

We were able to identify the relationship between sexual dysfunctions and other health conditions. Our study can be the basis for some form of screening and early assistance/intervention programs.

## Figures and Tables

**Table 1:** Characteristics of the participants.

Characteristics	Total *n* = 5942	FOD *n* = 547	FSIAD *n* = 100	GPPPD *n* = 276	Control *n* = 2120
Marital status:					
Single *n* (%)	1387 (23.3)	142 (26.0) ***	27 (27.0) *	50 (18.1)	351 (28.8)
Friend with benefits *n* (%)	492 (8.3)	45 (8.2) ***	6 (6.0) *	26 (9.4)	178 (20.2)
Married/partnership *n* (%)	4010 (67.6)	356 (65.2) ***	67 (67.0) *	196 (71.0)	1572 (74.2)
In more relationships (polyamory) *n* (%)	47 (0.8)	3 (0.5) ***	0 (0.0) *	4 (1.4)	17 (0.8)
Place of living:					
Village *n* (%)	1489 (25.1)	147 (26.9)	29 (29.0)	63 (22.8)	533 (21.1)
City/Town *n* (%)	3895 (65.6)	341 (62.3)	60 (60.0)	181 (65.6)	1404 (66.2)
Capital city *n* (%)	1557 (9.4)	59 (10.8)	11 (11.0)	32 (11.6)	183 (8.6)
Sexual history:					
Sexual education *n* (%)	4130 (69.5)	350 (64.0) ***	59 (59.0) **	177 (64.1) ***	1557 (73.4)
STD *n* (%)	846 (14.2)	103 (18.8) ***	16 (16.0)	51 (18.5) *	269 (12.7)
Sexual abuse *n* (%)	822 (13.8)	90 (16.5) ***	19 (19.0) *	54 (19.6) ***	229 (10.8)
Sexual orientation					
Heterosexual *n* (%)	5477 (92.2)	499 (91.2)	87 (87.0) *	251 (90.9) *	1977 (93.3)
Homosexual *n* (%)	68 (1.1)	4 (0.7)	2 (2.0) *	6 (2.2) *	24 (1.1)
Bisexual *n* (%)	377 (6.3)	44 (8.0)	10 (10.0) *	17 (6.2) *	118 (5.6)
Asexual *n* (%)	19 (0.3)	0 (0.0)	1 (1.0) *	2 (0.7) *	1 (0.0)

Significance of the difference between the individual sexual disorder groups and the control group. * *p* < 0.05, ** *p* <0.01, *** *p* < 0.001. STD: Sexually transmitted diseases.

**Table 2:** Differences between the FOD group and control group in sexual experience, age of first pornographic content, and satisfaction.

Variables	Group	Mean (SD)	Median	*n*	Mean Rank	Sum of Ranks	U	Effect Size (r)	*p* (2-Tailed)
Age (years)	FOD	22.12 (3.41)	22.0	547	1323.55	723,983.50	574,105.500	0.0069	0.720
	Control	22.26 (3.64)	21.0	2120	1336.70	2,833,794.50			
Evaluation of first sexual experience	FOD	5.20 (2.55)	5.0	520	1137.35	622,132.50	472,254.500	0.1305	<0.001
	Control	6.03 (2.55)	6.0	1929	1384.74	2,935,645.50			
Age of first pornographic content	FOD	14.01 (3.35)	14.0	547	1127.14	586,111.50	450,651.500	0.0692	<0.001
	Control	14.64 (3.07)	15.0	2120	1251.38	2,413,913.50			
Satisfaction with									
own sexual attraction	FOD	6.19 (2.07)	7.0	547	1116.35	610,645.50	460,767.500	0.1458	<0.001
	Control	6.92 (1.93)	7.0	2120	1390.16	2,947,132.50			
own body image	FOD	4.84 (2.63)	5.0	547	1117.87	611,474.00	461,596.000	0.1436	<0.001
	Control	5.79 (2.50)	6.0	2120	1389.77	2,946,304.00			
own genitalia	FOD	6.73 (2.58)	7.0	547	1020.51	558,217.50	408,339.500	0.2106	<0.001
	Control	7.99 (2.13)	9.0	2120	1414.89	2,999,560.50			

FOD: Female Orgasmic Disorder.

**Table 3:** Differences between the FSIAD group and control group in sexual experience, age of first pornographic content, and satisfaction.

Variables	Group	Mean (SD)	Median	*n*	Mean Rank	Sum of	U	Effect Size (r)	*p* (2-Tailed)
Age (years)	FSIAD	21.67 (3.19)	21.0	100	1026.54	102,654.00	97,604.000	0.0261	0.178
	Control	22.26 (3.64)	21.0	2120	1114.46	2,362,656.00			
Evaluation of first sexual experience	FSIAD	5.53 (2.55)	5.0	90	985.36	88,682.00	84,587.000	0.0390	0.066
	Control	6.03 (2.55)	6.0	2120	1110.60	2,354,473.00			
Age of the first pornographic content	FSIAD	14.15 (3.35)	14.0	93	926.52	86,166.00	81,795.000	0.0321	0.148
	Control	14.64 (3.07)	15.0	1929	1015.60	1,959,087.00			
Satisfaction with									
own sexual attraction	FSIAD	6.23 (2.07)	6.0	100	897.89	89,788.50	84,738.500	0.0668	<0.001
	Control	6.92 (1.93)	7.0	2120	1120.53	2,375,521.50			
own body image	FSIAD	4.79 (2.66)	4.5	100	876.89	87,688.50	82,638.500	0.0727	<0.001
	Control	5.79 (2.50)	6.0	2120	1121.52	2,377,621.50			
own genitalia	FSIAD	6.75 (2.54)	7.0	100	779.82	77,982.00	72,932.000	0.1044	<0.001
	Control	7.99 (2.13)	9.0	2120	1126.10	2,387,328.00			

FSIAD: Female Sexual Interest/Arousal Disorder.

**Table 4:** Differences between the GPPPD group and control group in sexual experience, age of first pornographic content, and satisfaction.

Variables	Group	Mean (SD)	Median	*n*	Mean Rank	Sum of Ranks	U	Effect Size (r)	*p* (2-Tailed)
Age (18-35)	GPPPD	22.16 (3.47)	22.0	276	1193.78	329,482.50	291,256.500	0.0024	0.452
	Control	22.26 (3.64)	21.0	2120	1199.11	2,542,123.50			
Evaluation of first sexual experience	GPPPD	4.92 (2.73)	5.0	276	951.94	262,736.50	224,510.500	0.1294	<0.001
	Control	6.03 (2.55)	6.0	2120	1230.60	2,608,869.50			
Age of the first pornographic content	GPPPD	14.18 (3.23)	15.0	251	1026.93	262,736.50	226,132.500	0.0366	0.087
	Control	14.64 (3.07)	15.0	1929	1098.77	2,119,531.50			
Satisfaction with									
own sexual attraction	GPPPD	6.03 (2.01)	6.00	276	911.82	251,661.00	213,435.000	0.1518	<0.001
	Control	6.92 (1.93)	7.00	2120	1235.82	2,619,945.00			
own body image	GPPPD	4.93 (2.45)	5.00	276	989.14	273,001.50	234,775.500	0.1099	<0.001
	Control	5.79 (2.50)	6.00	2120	1225.76	2,598,604.50			
own genitalia	GPPPD	6.78 (2.45)	7.00	276	869.90	240,093.50	201,867.500	0.1748	<0.001
	Control	7.99 (2.13)	9.00	2120	1241.28	2,631,512.50			

GPPPD: Genito-Pelvic Pain/Penetration Disorder.

**Table 5:** Differences between the MFSD group and control group in sexual experience, age of first pornographic content, and satisfaction.

Variables	Group	Mean (SD)	Median	*n*	Mean Rank	Sum of Ranks	U	Effect Size (r)	*p* (2-Tailed)
Age (years)	MFSD	22.16 (3.47)	22.00	288	1243.62	358,162.50	294,013.500	0.0208	0.306
	Control	22.26 (3.64)	21.00	2120	1199.19	2,542,273.50			
Evaluation of first sexual experience	MFSD	4.92 (2.73)	5.00	288	921.51	265,393.50	223,777.500	0.1510	<0.001
	Control	6.03 (2.55)	6.00	2120	1242.94	2,635,042.50			
Age of the first pornographic content	MFSD	14.18 (3.23)	15.00	269	1010.21	271,746.00	235,431.000	0.0528	0.012
	Control	14.64 (3.07)	15.00	1929	1111.95	2,144,955.00			
Satisfaction with									
own sexual attraction	MFSD	5.36 (2.32)	6.00	288	786.70	226,570.50	184,954.500	0.2247	<0.001
	Control	6.92 (1.93)	7.00	2120	1261.26	2,673,865.50			
own body image	MFSD	4.32 (2.72)	5.00	288	875.71	252,204.50	210,588.500	0.1755	<0.001
	Control	5.79 (2.57)	6.00	2120	1249.17	2,648,231.50			
own genitalia	MFSD	5.83 (2.71)	7.00	288	710.51	204,627.00	163,011.000	0.2668	<0.001
	Control	7.99 (2.13)	9.00	2120	1271.61	2,695,809.00			

MFSD: Multiple Female Sexual Dysfunction.

## Data Availability

Not applicable.

## References

[1] Rosen R., Brown C., Heiman J., Leiblum S., Meston C., Shabsigh R., Ferguson D., D’Agostino R. (2000). The Female Sexual Function Index (FSFI): A multidimensional self-report instrument for the assessment of female sexual function. J. Sex Marital Ther..

[2] Khajehei M., Doherty M., Tilley P.J. (2015). An update on sexual function and dysfunction in women. Arch. Womens Ment. Health.

[3] Goldstein I., Kim N.N., Clayton A.H., DeRogatis L.R., Giraldi AParish S.J., Pfaus J., Simon J.A., Kingsberg S.A., Meston C., Stahl S.M. (2017). Hypoactive Sexual Desire Disorder: International Society for the Study of Women’s Sexual Health (ISSWSH) Expert Consensus Panel Review. Mayo Clin. Proc..

[4] Stephenson K.R., Hughan C.P., Meston C.M. (2012). Childhood sexual abuse moderates the association between sexual functioning and sexual distress in women. Child. Abuse Negl..

[5] Wallwiener S., Strohmaier J., Wallwiener L.M., Schönfisch B., Zipfel S., Brucker S.Y., Rietschel M., Wallwiener C.W. (2016). Sexual Function Is Correlated With Body Image and Partnership Quality in Female University Students. J. Sex. Med..

[6] Reissing E.D., Binik Y.M., Khalifé S., Cohen D., Amsel R. (2003). Etiological correlates of vaginismus: Sexual and physical abuse, sexual knowledge, sexual self-schema, and relationship adjustment. J. Sex. Marital. Ther..

[7] Pazmany E., Bergeron S., Van Oudenhove L., Verhaeghe J., Enzlin P. (2013). Aspects of sexual self-schema in premenopausal women with dyspareunia: Associations with pain, sexual function, and sexual distress. J. Sex. Med..

[8] Stephenson K.R., Meston C.M. (2015). Why is impaired sexual function distressing to women? The primacy of pleasure in female sexual dysfunction. J. Sex. Med..

[9] Safarinejad M.R. (2006). Female sexual dysfunction in a population-based study in Iran: Prevalence and associated risk factors. Int. J. Impot. Res..

[10] Brassil D.F., Keller M. (2002). Female sexual dysfunction: Definitions, causes, and treatment. Urol. Nurs..

[11] McCabe M.P., Sharlip I.D., Lewis R., Atalla E., Balon R., Fisher A.D., Laumann E., Lee S.W., Segraves R.T. (2016). Incidence and Prevalence of Sexual Dysfunction in Women and Men: A Consensus Statement from the Fourth International Consultation on Sexual Medicine 2015. J. Sex. Med..

[12] Nicolosi A., Laumann E.O., Glasser D.B., Moreira E.D., Paik A., Gingell C. (2004). Global Study of Sexual Attitudes and Behaviors Investigators’ Group. Sexual behavior and sexual dysfunctions after age 40: The global study of sexual attitudes and behaviors. Urology.

[13] Starc A., Jukić T., Poljšak B., Dahmane R. (2018). Female Sexual Function and Dysfunction: A Cross-National Prevalence Study in Slovenia. Acta Clin. Croat..

[14] American Psychiatric Association (2013). Diagnostic and Statistical Manual of Mental Disorders.

[15] Clayton A.H., Valladares Juarez E.M. (2019). Female Sexual Dysfunction. Med. Clin. N. Am..

[16] Fugl-Meyer K.S., Oberg K., Lundberg P.O., Lewin B., Fugl-Meyer A. (2006). On orgasm, sexual techniques, and erotic perceptions in 18- to 74-year-old Swedish women. J. Sex. Med..

[17] Laumann E.O., Nicolosi A., Glasser D.B., Paik A., Gingell C., Moreira E., Wang T., GSSAB Investigators’ Group (2005). Sexual problems among women and men aged 40-80 y: Prevalence and correlates identified in the Global Study of Sexual Attitudes and Behaviors. Int. J. Impot. Res..

[18] Laumann E.O., Paik A., Rosen R.C. (1999). Sexual dysfunction in the United States: Prevalence and predictors. JAMA.

[19] Lewis R.W., Fugl-Meyer K.S., Bosch R., Fugl-Meyer A.R., Laumann E.O., Lizza E., Martin-Morales A. (2004). Epidemiology/risk factors of sexual dysfunction. J. Sex. Med..

[20] Hayes R.D., Bennett C.M., Fairley C.K., Dennerstein L. (2006). What can prevalence studies tell us about female sexual difficulty and dysfunction?. J. Sex. Med..

[21] Latthe P., Latthe M., Say L., Gülmezoglu M., Khan K.S. (2006). WHO systematic review of prevalence of chronic pelvic pain: A neglected reproductive health morbidity. BMC Public Health.

[22] Peixoto M.M., Nobre P. (2015). Prevalence and sociodemographic predictors of sexual problems in Portugal: A population-based study with women aged 18 to 79 years. J. Sex. Marital Ther..

[23] Smith A.M., Lyons A., Ferris J.A., Richters J., Pitts M.K., Shelley J.M., Simpson J.M., Heywood W., Patrick K. (2012). Incidence and persistence/recurrence of women’s sexual difficulties: Findings from the Australian Longitudinal Study of Health and Relationships. J. Sex. Marital Ther..

[24] Barlow D.H., Cardozo L.D., Francis R.M., Griffin M., Hart D.M., Stephens E., Sturdee D.W. (1997). Urogenital ageing and its effect on sexual health in older British women. Br. J. Obstet. Gynaecol..

[25] Villeda Sandoval C.I., Calao-Pérez M., Enríquez González A.B., Gonzalez-Cuenca E., Ibarra-Saavedra R., Sotomayor M., Castillejos Molina R.A. (2014). Orgasmic dysfunction: Prevalence and risk factors from a cohort of young females in Mexico. J. Sex. Med..

[26] Graham C.A. (2010). The DSM diagnostic criteria for female orgasmic disorder. Arch. Sex. Behav..

[27] Bleck R.T., Loveless J. (1987). Human sexual malfunction: A consideration of ‘inner- mind’ thought process. Int. J. Psychosom..

[28] Bradford A. Female Orgasmic Disorder: Epidemiology, Pathogenesis, Clinical Manifestations, Course, Assessment, and Diagnosis. UpToDate. https://www.uptodate.com/contents/female-orgasmic-disorder-epidemiology-pathogenesis-clinical-manifestations-course-assessment-and-diagnosis.

[29] Rellini A. (2008). Review of the empirical evidence for a theoretical model to understand the sexual problems of women with a history of CSA. J. Sex. Med..

[30] Leonard L.M., Follette V.M. (2002). Sexual functioning in women reporting a history of child sexual abuse: Review of the empirical literature and clinical implications. Annu. Rev. Sex. Res..

[31] Alizadeh A., Farnam F., Raisi F., Parsaeian M. (2019). Prevalence of and Risk Factors for Genito-Pelvic Pain/Penetration Disorder: A Population-Based Study of Iranian Women. J. Sex. Med..

[32] Mitchell K.R., Geary R., Graham C.A., Datta J., Wellings K., Sonnenberg P., Field N., Nunns D., Bancroft J., Jones K.G. (2017). Painful sex (dyspareunia) in women: Prevalence and associated factors in a British population probability survey. BJOG Int. J. Obstet. Gynaecol..

[33] Ramezani Tehrani F., Farahmand M., Simbar M., Malek Afzali H. (2014). Factors associated with sexual dysfunction; a population based study in Iranian reproductive age women. Arch. Iran Med..

